# Modification of the Spectral Absorbance Difference Method for Determining the Dissociation Constants of Polyprotic Acids with Delocalized π‑Systems

**DOI:** 10.1021/acsomega.5c06049

**Published:** 2025-10-01

**Authors:** Huy Do, Galina Z. Goloverda, Vladimir L. Kolesnichenko

**Affiliations:** Chemistry Department, 5785 Xavier University of Louisiana, 1 Drexel Dr., New Orleans, Louisiana 70125, United States

## Abstract

Polyprotic organic acids with suitable molecular geometry and acidic properties are frequently employed in the development of novel nano- and macroscale materials. These acids can bind to inorganic surfaces in a bridging mode, effectively facilitating junctions at organic–inorganic interfaces. This study was motivated by the potential of triprotic 2-hydroxyisophthalic (tenacic) acid and its derivatives to fulfill this role. The absorbance spectra of the unsubstituted tenacic and 4-nitrotenacic acids were found to be strongly pH-dependent. This behavior is attributed to varying degrees of electron delocalization in the π-systems of the conjugate bases. The previously reported spectral absorbance difference method was applied in this work without modification to determine the first dissociation constants of both acids. To determine the second dissociation constants for both acids and the third for 4-nitrotenacic acid, we modified the method to improve the accuracy of the measured *K*_b_ of their conjugate bases. Utility of this method may be extended to other compounds that exhibit pH-dependent variations in π-electron delocalization.

## Graphical abstract

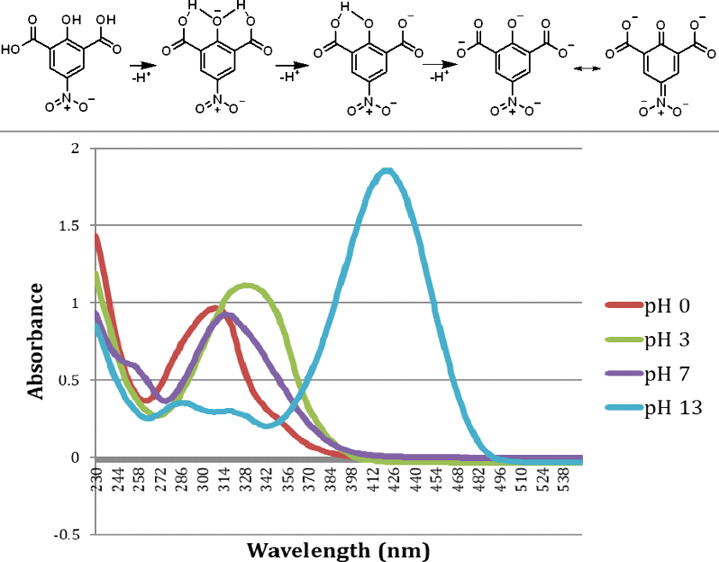

## Introduction

2-Hydroxyisophthalic acid, further referred to as “tenacic” acid, and its derivatives exhibit remarkable properties that make them highly attractive for the development of novel materials. The three donor groups in the 2-hydroxyisophthalate moiety allow for diverse modes of metal binding, including bridging and chelation as well as double chelation in an M_2_L_2_ core. This flexibility enables the formation of a variety of metal–organic framework (MOF) motifs, characterized by spin interactions between neighboring metal ions and involvement of the ligand’s π-system.^1–13^

Among the heteroatom-free carboxylic acids, tenacic acid is relatively strong, with its first p*K*_a_ reported in the range of 1.6–2.1.^14,15^ Notably, its most acidic site is not one of its carboxyl groups but the phenolic hydroxyl group due to a particularly strong internal hydrogen bond. Crystal structure studies reveal that the 2-hydroxyisophthalate monoanion is nearly planar and highly symmetrical about the axis passing through phenolic oxygen and carbon atom para-positioned to it (Scheme 1a). Interestingly, both OH protons are closer to the carboxylic oxygens, with the H–O (phenolic) bond distance being nearly 62% longer than the H–O (carboxylic) bond distance.^16^

**Scheme 1:**
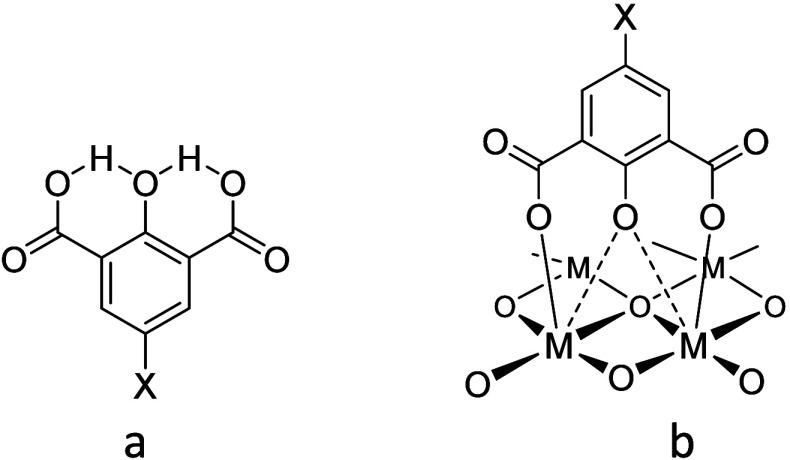
(a) Internal Hydrogen Bonding in the Tenacate Monoanion; (b) A Hypothetical Binding Mode of 2-Hydroxyisophthalic Acid to the Solid Metal Oxide Surface

According to our preliminary crystal structure results,[Fn fn1] the fully deprotonated tenacate ligand is present in a double-chelated Mg_2_L_2_ core (L = tenacate^3–^) of the anionic coordination complex synthesized by the following reaction: 3Mg + 2H_3_L + 10H_2_O → Mg­(OH_2_)_6_[Mg_2_L_2_(OH_2_)_4_] + 3H_2_ (to be published elsewhere).

Additional notable properties of tenacic acid include its high fluorescence and its ability to sensitize lanthanide ions.^9,10^ It also demonstrates a strong affinity to metal oxide surfaces, earning its “tenacic” name due to this property (Scheme 1b).^17^ These characteristics make tenacic acid an excellent candidate for constructing novel materials, including medical theranostic agents, dye- and quantum-dot-sensitized solar cells, organic LEDs, sensors, and supramolecular structures with unique magnetic and optical properties.

Understanding structural trends, bonding, spectroscopic characteristics, and reactivity of primary precursors or intermediates is essential for advancing research and materials development. This need served as the primary motivation for our current study.

Here, we report the synthesis and spectrophotometric p*K*_a_ determination of the parent compound (X = H) and its nitro derivative (X = NO_2_), as shown in Scheme 1a. Our modification of the absorbance difference spectral method enabled the determination of the first two p*K*_a_ values for X = H and all three p*K*_a_ values for X = NO_2_. The results are discussed in the context of the molecular structure, resonance effects, and intramolecular hydrogen bonding.

## Results and Discussion

The absorbance spectra of unsubstituted tenacic acid (acid 1) and 4-nitrotenacic acid (acid 2), shown in Figures 1 and 2, exhibit strong pH dependence, enabling the use of the absorbance difference spectral method for their p*K*_a_ determination^18,19^ and its modification presented in this work.

**Figure 1:**
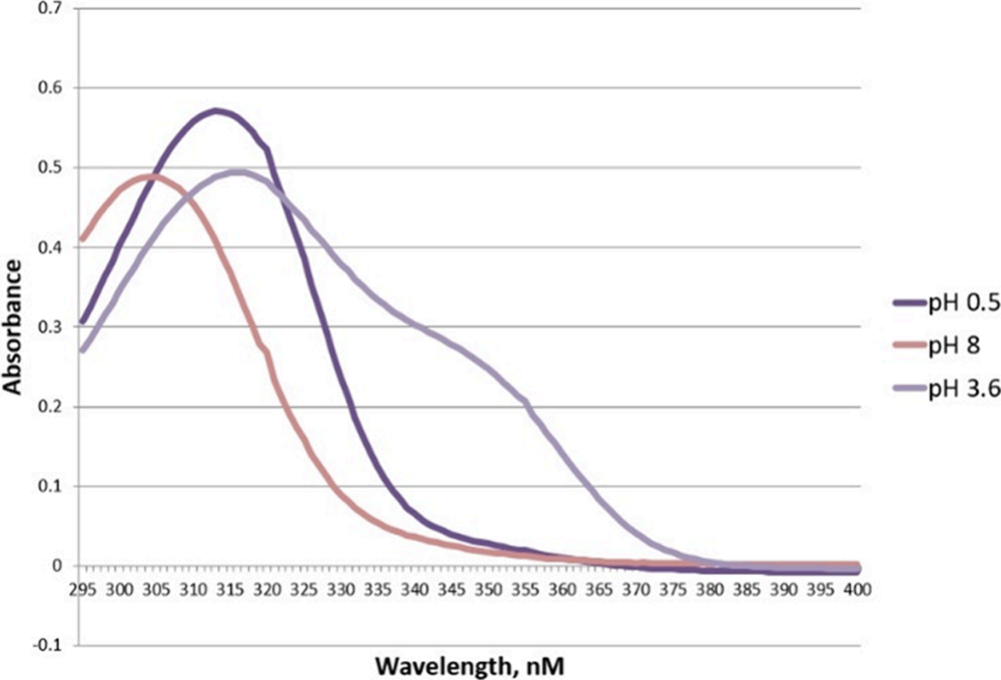
Full-scale spectra of tenacic acid (acid 1) in three forms, H_3_A, H_2_A^–^, and HA^2–^.

**Figure 2:**
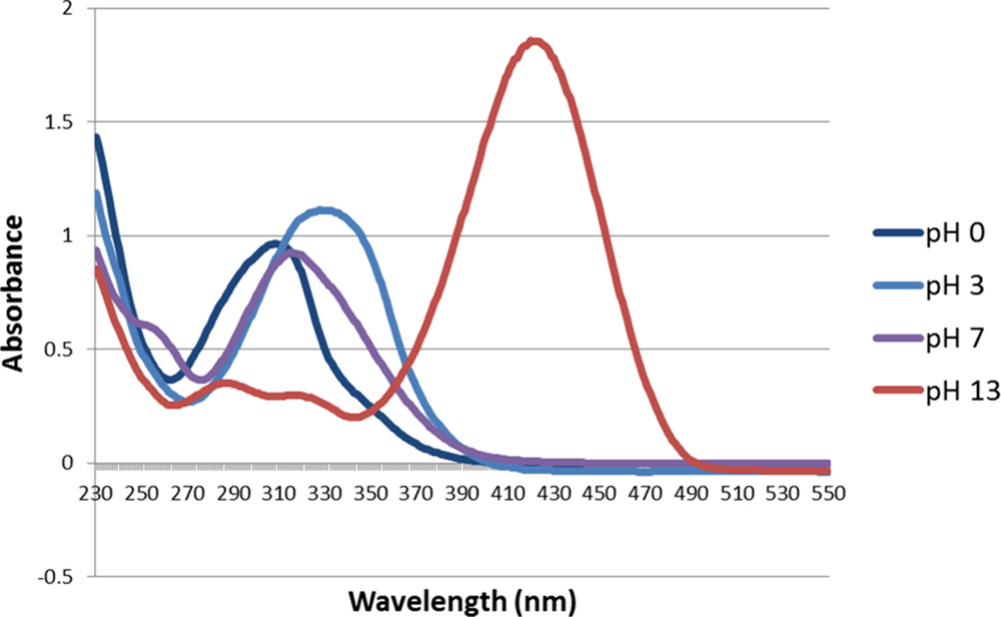
Full-scale spectra of 4-nitrotenacic acid (acid 2) in four forms, H_3_A, H_2_A, HA^2–^, and A^3–^.

To determine p*K*_a1_, spectra of both undissociated acids 1 and 2 were recorded at the lowest pH and used as references. These reference spectra were compared with spectra recorded at progressively higher pH values, where the concentration of the monoanion increased (but the overall analyte concentration was maintained the same). Data analysis was performed using Microsoft Excel. Detailed explanations and plots are provided below for acid 1, and corresponding plots for acid 2 are available in the Supporting Information (SI).

For tenacic acid, four sets of spectra were collected at fixed analyte concentrations (0.1, 0.2, 0.3, and 0.4 mM). Each set included measurements at five pH values: 0.5, 1.5, 2.0, 2.5, and 3.62. An example set of spectra at 0.1 mM is shown in Figure 3. Differential spectra (Figure 4) were obtained by subtracting the reference spectrum (pH = 0.5) from each spectrum within the same set recorded at higher pH values. Similar sets of spectra for 4-nitrotenacic acid are presented in Figures S1 and S2. Peaks and troughs in the differential spectra were used to identify analytical wavelengths (Table 1). A set of absolute absorbance differences is observed at these wavelengths (Tables S1 and S2), plotted against analyte concentration at each fixed pH, yielded straight-line graphs (Figure 5 and Figure S3).

**Figure 3:**
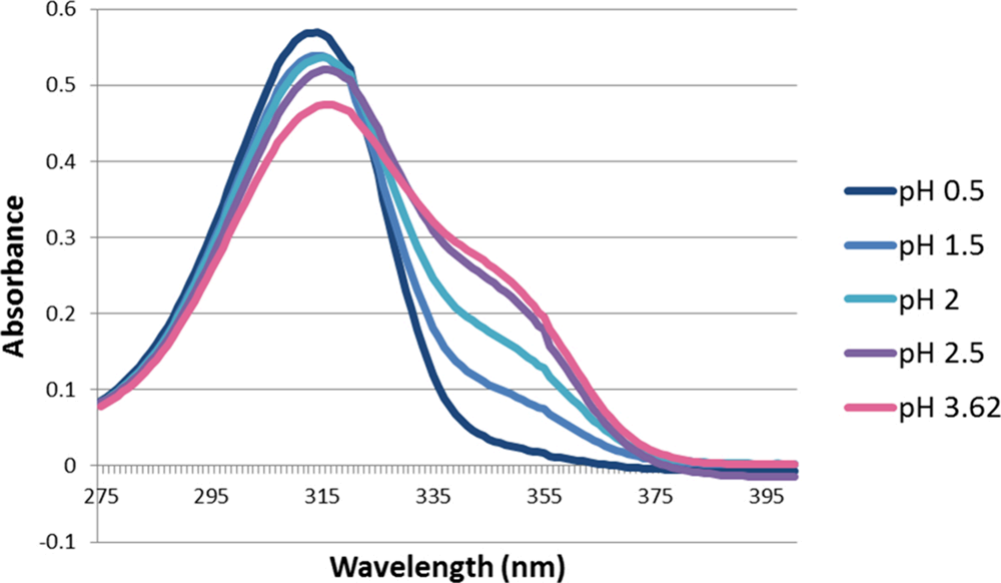
Absorbance spectra of 0.1 mM tenacic acid at equilibrium of H_3_A ⇄ H_2_A^–^. Similar sets of curves were obtained for 0.2, 0.3, and 0.4 mM samples at the same pH as here.

**Figure 4:**
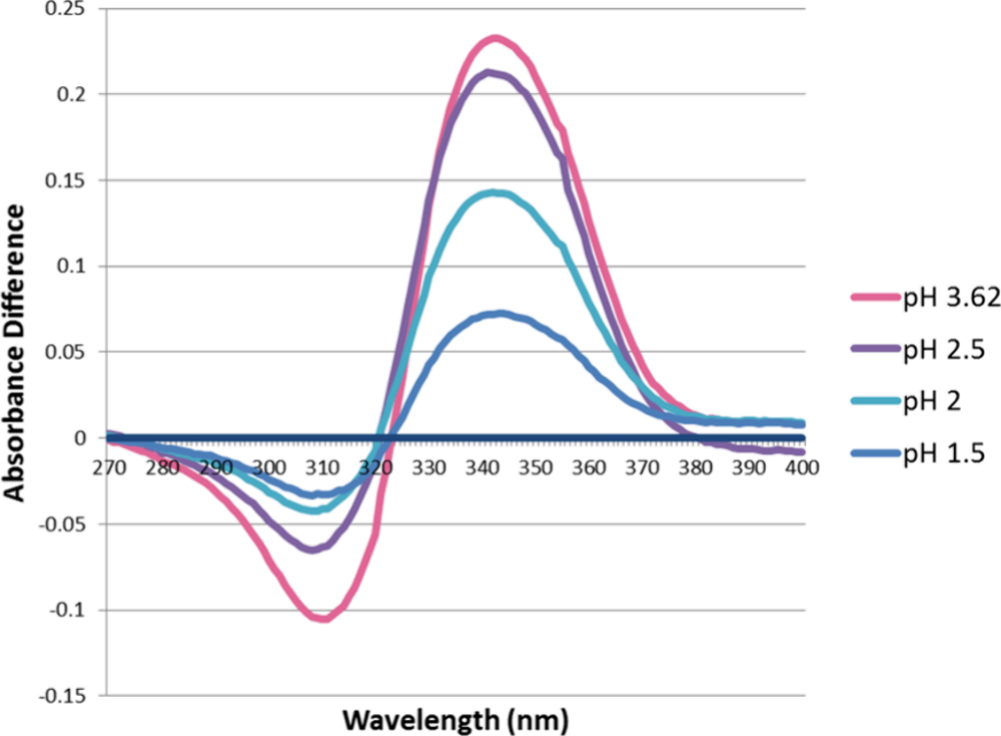
Absorbance difference spectra of 0.1 mM tenacic acid at equilibrium H_3_A ⇄ H_2_A^–^. Similar sets of curves were obtained for 0.2, 0.3, and 0.4 mM samples at the same pH as here.

**Table 1:** Analytical Wavelengths, nm, Used for Determination of p*K*_a_’s of Acids 1 and 2

acid	p*K*_a1_	p*K*_a2_	p*K*_a3_
1	342 and 310	330 and 295	
2	340 and 286	344 and 260	422 and 316

**Figure 5:**
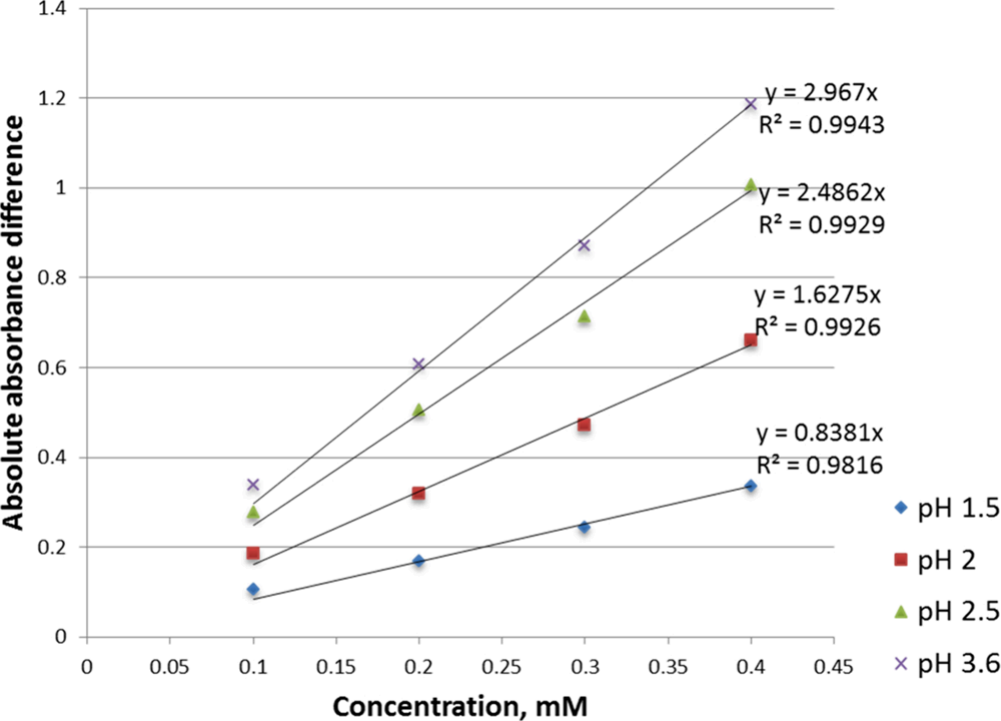
Absolute absorbance difference vs concentration for tenacic acid at the equilibrium H_3_A ⇄ H_2_A^–^. The absorbance difference values were obtained at analytical wavelengths of 342 nm (maximum) and 310 nm (minimum).

The *K*_a_ values were calculated using eq 1 ^19^ based on the slopes of two selected lines at a time.

$$K_{a}=\frac{[H1]slope1-[H2]slope2}{slope2-slope1}$$ 1

The standard deviation was calculated from six p*K*_a1_ values obtained for both acids using different pairs of lines.[Fn fn2]

To determine p*K*_a2_, the method was modified to account for uncertainties in the spectra of pure representative species. Instead of using the spectrum of the monoanion (H_2_A^–^) as a reference and comparing it with spectra at higher pH values, we selected the spectrum of the dianion (HA^2^^–^), which was easier to identify (at pH = 8 for acid 1). This spectrum was then compared with those recorded at lower pH values (5.5, 5.0, 4.5, and 4.0), where the concentration of H_2_A^–^ progressively increased (Figure S4). Differential spectra (Figure S5) were obtained by subtracting the reference spectrum (pH = 8.0) from each spectrum within the same set of concentrations recorded at lower pH values. A set of absolute absorbance differences at wavelengths of 330 and 295 nm (Table S3), plotted against analyte concentration for each fixed pH, produced straight-line graphs (Figure S6).

The slopes obtained from the absolute absorbance difference plots were used to calculate the *K*_b_ values according to eq 2 (note: concentration of OH^–^ was used here). These values were then converted to the corresponding *K*_a_ values.[Fn fn3]

$$K_{b}=\frac{[OH1]slope1-[OH2]slope2}{slope2-slope1}$$ 2

Similarly, for p*K*_a3_, the spectrum of the trianion (A^3^^–^) (well-defined for 4-nitrotenacic acid only, see Figure 2) was identified and compared with spectra at lower pH values, where the concentration of HA^2^^–^ progressively increased (Figures S7–S9).

The acidity constants determined by using the spectrophotometric method at *T* = 22 °C are summarized in Table 2.

**Table 2:** Acid Dissociation Constants for 1 and 2

	tenacic acid (1)	4-nitrotenacic acid (2)
p*K*_a1_	1.92 ± 0.04	0.89 ± 0.02
p*K*_a2_	4.57 ± 0.01	4.05 ± 0.29
p*K*_a3_		11.12 ± 0.01

Optimal concentrations of our samples being in the range of 0.1 to 0.4 mmol/L for tenacic acid and 0.02 to 0.1 mmol/L for 4-nitrotenacic acid provided that absorbances change linearly with concentration (Figures S10 and S11).

The standard deviations for the determined p*K*_a_ values are generally small, with the exception of the p*K*_a__2_ of 4-nitrotenacic acid, which shows higher variability. This increased uncertainty is likely due to partial overlap of the dissociation equilibria in its solutions. Additionally, the insufficient spectral distinction between the dianion and trianion forms of acid 1 hindered the determination of its p*K*_a__3_ using the absorption difference spectral method.

As shown in Table 2, the first p*K*_a_ of 4-nitrotenacic acid is more than one pH unit lower than that of the unsubstituted acid, while the second p*K*_a_ is less significantly affected. This trend aligns with the well-established enhancement of acid dissociation in nitrophenols relative to their parent phenols, particularly when nitro groups occupy ortho or para positions relative to the phenolic hydroxyl group. For instance, the p*K*_a_ values of 2,4-dinitrophenol (4.09) and 3,5-dinitrophenol (6.68) differ by more than 2.5 orders of magnitude. This observation further supports the previously stated conclusion^16^ that the most acidic proton in both tenacic and 4-nitrotenacic acids is phenolic.

The absorption spectrum of the 4-nitrotenacate trianion differs significantly from that of its dianion (Figure 2); this is likely due to negative charge delocalization in the quinoid resonance form involving the nitro group. Intramolecular hydrogen bonding in the dianion, however, may stabilize its phenolate form, disfavoring the same type of resonance (Scheme 2).

**Scheme 2:**
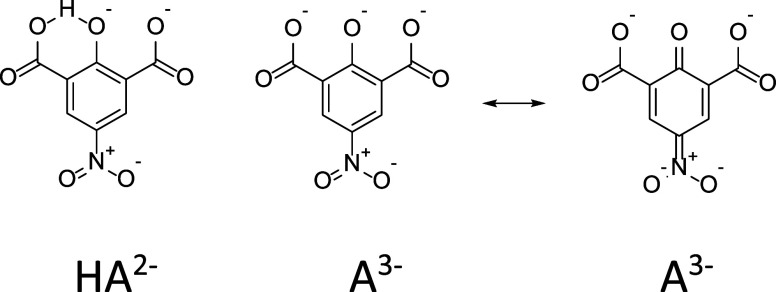
Structure of the 4-Nitrotenacate Dianion and of the Two Resonance Forms of the Trianion

The potentiometric titration method is more commonly used for p*K*_a_ determination than the spectrophotometric method due to its simplicity and shorter duration, despite its limitations in analyte concentration, p*K*_a_ range and sensitivity.^20^ To enable a direct comparison, we conducted potentiometric titrations alongside spectrophotometric measurements for both acids (1) and (2).

The potentiometric method could directly determine only p*K*_a__2_, as p*K*_a__1_ and p*K*_a__3_ for both acids fell outside the potentiometer’s working range – being too low and too high, respectively. Moreover, the low water solubility of the unsubstituted acid (1) (2.6 g/L at 22 °C compared to 17.5 g/L for acid 2) further hindered the determination of its p*K*_a__1_.

The values of p*K*_a__1_ (for acid 2) and p*K*_a__3_ (for both acids 1 and 2) were obtained using an indirect method for processing the titration data.^21^ Details of this procedure are provided in the Supporting Information.

The results obtained for both acids using both methods are summarized in Table 3. As shown, the p*K*_a_ values determined by the potentiometric method (uncorrected) show moderate deviation from the spectrophotometric values that one could expect. The spectrophotometric p*K*_a2_ value (4.57 ± 0.01) determined in this study for acid 1 aligns well with published values of 4.53 ^14^ and 4.50,^15^ in contrast to the potentiometric result (4.72). These findings clearly indicate that the spectrophotometric method yields more accurate results than the potentiometric approach.

**Table 3:** Acid Dissociation Constants for Acids 1 and 2 Determined by Spectrophotometric (S) and Potentiometric (P) Methods

	tenacic acid		4-nitrotenacic acid	
	S	P	S	P
p*K*_a1_	1.92 ± 0.04		0.89 ± 0.02	0.72
p*K*_a2_	4.57 ± 0.01	4.72	4.05 ± 0.29	4.23
p*K*_a3_		11.11	11.12 ± 0.01	10.69

## Experimental Section

### General

The pH measurements were done with a SympHony VWR SB21 pH meter and Ag/AgCl combination glass electrode. Two-point calibrations were done using standard pH 4, pH 7, and pH 10 buffers. Stable pH was maintained for each sample by the universal pH 1.8–12 buffer composed of 0.04 M phosphoric, acetic, and boric acids each, plus a variable amount of 0.2 M NaOH (0 to 50 vol %). The samples with extreme pH beyond the range of this buffer were prepared using 1.00 M NaOH or HCl. The UV–vis spectra were obtained on a Beckman DU-800 spectrometer in 10 mm quartz cells. The pH titrations were done at 22 °C, and the temperature compensation was done manually. Analyte concentrations ranged 7 × 10^–3^ to 8 × 10^–3^ M for (1) and 5.6 × 10^–3^ to 1.1 × 10^–2^ M for (2); the titrant was 0.0990 M NaOH.

### 2-Hydroxyisophthalic (Tenacic) Acid (Acid 1)

A 250 mL stainless steel beaker was charged with 120 g (1.62 mol) of the granular KOH·H_2_O and 25 mL of water. After cooling for 5 min, 20.0 g (0.13 mol) of 3-methyl salicylic acid was added to the solution gradually while stirring with nickel spatula followed by 120 g (0.50 mol) of PbO_2_. The resulting mixture was flame-heated while intensively stirring with a Bunsen burner. During 10–15 min heating session, the mixture turned thicker first and then softened, then liquefied, briefly boiled, and changed its color from black to red. Heating was continued until the melt became free-flowing and Pb_3_O_4_ formed as red crystals. The reaction mixture was allowed to cool; the solidifying melt was loosened by stirring. After cooling, the solid was treated with 300 mL of deionized water and stirred until the KOH melt dissolved and Pb_3_O_4_ separated from the solution. The crystalline red Pb_3_O_4_ fraction was separated by decantation, and the yellow-orange microcrystalline fraction was separated by brief centrifuging. The precipitates were washed with additional 2 × 50 mL of water, and all aqueous solutions were combined. The resulting solution was acidified with a solution of sulfuric acid: 51 mL of concentrated H_2_SO_4_ in 100 mL of water. Addition of sulfuric acid was continued until the pH dropped to 7–8; the precipitation of PbSO_4_ at this point was complete. The precipitated lead sulfate was separated by centrifuging and rinsed with water to improve the yield of the product. The separated supernatant solution was further acidified with the remaining sulfuric acid; this caused precipitation of the target product (1). After cooling in an ice bath, the solid was filtered off on a medium glass frit, washed with 0.1 M HCl until a drop test with BaCl_2_ solution was negative and then with icy water, and finally transferred in a dish and air-dried. The typical yield of an off-white powder was 80–85%. Solid product was recrystallized from hot water and isolated as monodydrate off-white needles with mp 243–245 °C (after losing crystallization water at 100 °C). Anal. calc’d for C_8_H_6_O_5_·H_2_O: C, 48.00; H, 4.04. Found: C, 48.35; H, 4.02. HRMS (ESI) calcd for C_8_H_6_O_5_ [M - H] 181.0142, found 181.0139. ^1^H NMR (400 MHz, DMSO-*d*_6_) δ 7.93 (d, *J* = 7.6 Hz, 2H), 6.85 (t, *J* = 7.6 Hz, 1H).

### 4-Nitrotenacic Acid (Acid 2)

Tenacic acid monohydrate (10 mmol, 2.00 g) was dissolved in 20 mL of concentrated sulfuric acid (*d* = 1.83 g/cm^3^). The solution was rapidly stirred and heated until the temperature reached 120 °C and cooled down naturally. Starting at ambient temperature, the solution of potassium nitrate (15 mmol, 1.52 g) in 10 mL of concentrated sulfuric acid was added to solution of tenacic acid dropwise while stirring. The addition rate was adjusted so that the temperature did not rise higher than 35 °C. After mixing was complete, the solution was left overnight at ambient temperature. Next, the reaction solution was added to ∼100 g of crushed ice slowly while shaking. The precipitate of the product was filtered and washed with ∼100 mL of ∼4 M hydrochloric acid until the BaSO_4_ test turned negative. The product was transferred into a wide dish and dried in vacuum desiccator over NaOH. The typical yield of pale-yellow powder was 85–90%. Crystalline monohydrate was obtained by recrystallization from hot water. Anal. calc’d for C_8_H_5_NO_7_·H_2_O: C, 39.19; H, 2.88; N, 5.71. Found: C, 39.48; H, 2.57; N, 5.48. ^1^H NMR (400 MHz, DMSO-*d*_6_) δ 8.68 (s). ESI-MS calcd for C_8_H_5_NO_7_ [M-H] 225.9993, found 225.9988.

## Conclusions

The absorbance difference method and its modified version enabled accurate determination of the first two dissociation constants for tenacic acid and all three for 4-nitrotenacic acid. Among heteroatom-free carboxylic acids, tenacic acid is relatively strong, with a p*K*_a__1_ of 1.92. This acidity is attributed to the high stability of its monoanion, resulting from strong intramolecular hydrogen bonding around the phenolate oxygen atom. In contrast, the first dissociation constant of 4-nitrotenacic acid is significantly higher (p*K*_a__1_ = 0.89), consistent with known trends for ortho- and para-substituted nitrophenols. The strong donor properties and optimal alignment of coordinating groups in tenacic acid also contribute to its (and its derivatives’) affinity for metal oxide surfaces. These measurements were made possible by the strong pH dependence of the absorbance spectra of both tenacic and 4-nitrotenacic acids, which is linked to differences in electron delocalization within the π-systems of their conjugate bases. The approach presented here may be extended to other compounds that exhibit pH-dependent variations in π-electron delocalization.

## Supplementary Material













## Footnotes

◊ Crystal structure was determined by Alexander Nazarenko at Buffalo State College.

§ Calculations of p*K*_a1_ of tenacic acid (1) can be seen in the TA p*K*_a1_ excel file (as a part of the SI).

‡ Calculations of p*K*_a2_ of 4-nitrotenacic acid (2) can be seen in the NTA p*K*_a2_ excel file (as a part of the SI).

## Notes

Table S1: Absorbance difference data for determining p*K*_a1_ of tenacic acid; Figure S1: Absorbance spectra of 0.08 mM 4-nitrotenacic acid; Table S2: Absorbance difference data for determining p*K*_a1_ of 4-nitrotenacic acid; Figure S3: Absolute absorbance difference vs concentration for 4-nitrotenacic acid; Figure S4: Absorbance spectra of 0.4 mM tenacic acid; Figure S5: Absorbance difference spectra of 0.4 mM tenacic acid; Table S3: Absorbance difference data for determining p*K*_a2_ of tenacic acid; Figure S6: Absolute absorbance difference vs concentration for tenacic acid; Figure S7: Absorbance spectra of 0.10 mM 4-nitrotenacic acid; Figure S8: Absorbance difference spectra of 0.10 mM 4-nitrotenacic acid; Table S4: Absorbance difference data for determining p*K*_a3_ of 4-nitrotenacic acid; Figure S9: Absolute absorbance difference vs concentration for 4-nitrotenacic acid; Figure S10: Absorbance vs concentration for tenacic acid at pH 4.0 and at 312 nm maximum; Figure S11: Absorbance vs concentration for 4-nitrotenacic acid at pH 13 and at 422 nm maximum; Table S5: p*K*_a_ values for both 1 and 2 determined by potentiometric method (PDF)
TA p*K*_a1_ (XLSX)
NTA p*K*_b__2_ (XLSX)
TA p*K*_b__2_ (XLSX)
NTA p*K*_a1_ (XLSX)
NTA p*K*_b3_ (XLSX)
