# Supplementary material for: Modification of the Spectral Absorbance Difference Method for Determining the Dissociation Constants of Polyprotic Acids with Delocalized π‑Systems
Source: ACS Omega. 2025 Oct 1;10(40):47210–5. doi: 10.1021/acsomega.5c06049 (PMC12529155; doi:10.1021/acsomega.5c06049)
Supplement: Supplementary file 1 [file ao5c06049_si_001.pdf]

Modification of the Spectral Absorbance Difference Method for Determining the Dissociation Constants of Polyprotic Acids with Delocalized π-Systems

Huy Do, Galina Z. Goloverda * and Vladimir L. Kolesnichenko *

Xavier University of Louisiana, Chemistry Department, 1 Drexel Dr., New Orleans, LA 70125

SUPPLEMENTARY INFORMATION

Contents

2 – Table S1, Figure S1

3 – Figure S2, Table S2

4 – Figure S3

5 – Figure S4, Figure S5

6 – Table S3, Figure S6

7 – Figure S7, Figure S8

8 – Table S4, Figure S9

9 – Figure S10, Figure S11

10 – Potentiometric titration method

Table S1. Absorbance difference data for determining pK_{a1} of tenacic acid. The reference spectrum was for the sample with pH 0.5. Spectral maximum (Max) = 342 nm; spectral minimum (Min) = 310 nm. The absolute absorbance difference values (ΔA at Max – ΔA at Min) were used for plotting their dependence on concentration and pH.

C, mM		pH 3.62	pH 2.5	pH 2	pH 1.5
0.1	ΔA at Max	0.2324	0.2127	0.1433	0.0723
	ΔA at Min	-0.1054	-0.065	-0.0427	-0.0334
	ΔA at Max – ΔA at Min	0.3378	0.2777	0.186	0.1057
0.2	ΔA at Max	0.4427	0.3725	0.2452	0.126
	ΔA at Min	-0.1637	-0.1339	-0.0733	-0.0426
	ΔA at Max – ΔA at Min	0.6064	0.5064	0.3185	0.1686
0.3	ΔA at Max	0.6546	0.5451	0.3622	0.1942
	ΔA at Min	-0.2155	-0.1693	-0.1102	-0.0495
	ΔA at Max – ΔA at Min	0.8701	0.7144	0.4724	0.2437
0.4	ΔA at Max	0.8655	0.7439	0.4985	0.2423
	ΔA at Min	-0.3195	-0.2623	-0.1621	-0.0928
	ΔA at Max – ΔA at Min	1.185	1.0062	0.6606	0.3351

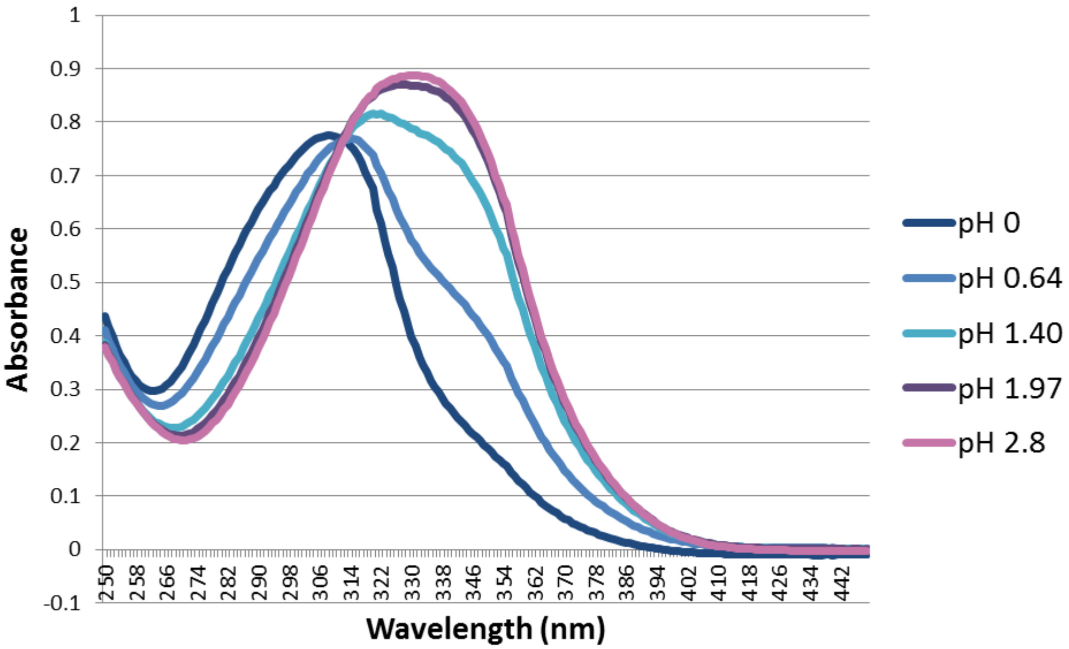

Figure S1. Absorbance spectra of 0.08 mM 4-nitrotenacic acid at equilibrium $H_3A \leftrightarrows H_2A^-$. Similar sets of spectra were obtained for 0.04, 0.06 and 0.10 mM samples at the same pH as here.

2

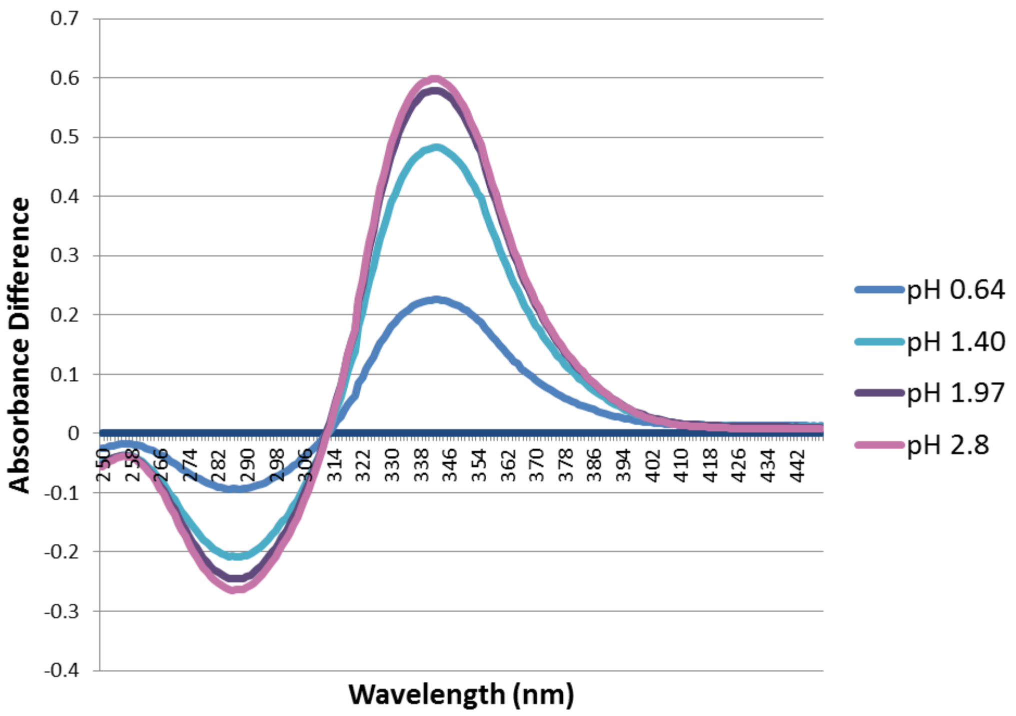

Figure S2. Absorbance difference spectra of 0.08 mM 4-nitrotenacic acid at equilibrium $H_3A \leftrightarrows H_2A^-$. Similar sets of curves were obtained for 0.04, 0.06 and 0.10 mM samples at the same pH as here.

Table S2. Absorbance difference data for determining pK_{a1} of 4-nitrotenacic acid. The reference spectrum was for the sample with pH 0. Spectral maximum (Max) = 340 nm; spectral minimum (Min) = 286 nm. The absolute absorbance difference values (ΔA at Max – ΔA at Min) were used for plotting their dependence on concentration and pH.

C, mM		pH 2.8	pH 2.0	pH 1.4	pH 0.6
0.04	ΔA at Max	0.2910	0.2899	0.2391	0.1102
	ΔA at Min	-0.1286	-0.1169	-0.0982	-0.0457
	ΔA at Max – ΔA at Min	0.4196	0.4068	0.3373	0.1559
0.06	ΔA at Max	0.4330	0.4308	0.3623	0.1665
	ΔA at Min	-0.1978	-0.1885	-0.1493	-0.0738
	ΔA at Max – ΔA at Min	0.6308	0.6193	0.5116	0.2403
0.08	ΔA at Max	0.5951	0.5771	0.4793	0.2241
	ΔA at Min	-0.2647	-0.2454	-0.2073	-0.0935
	ΔA at Max – ΔA at Min	0.8598	0.8225	0.6866	0.3176
0.10	ΔA at Max	0.7546	0.7018	0.5713	0.2709
	ΔA at Min	-0.3144	-0.3096	-0.2541	-0.1086
	ΔA at Max – ΔA at Min	1.069	1.0114	0.8254	0.3795

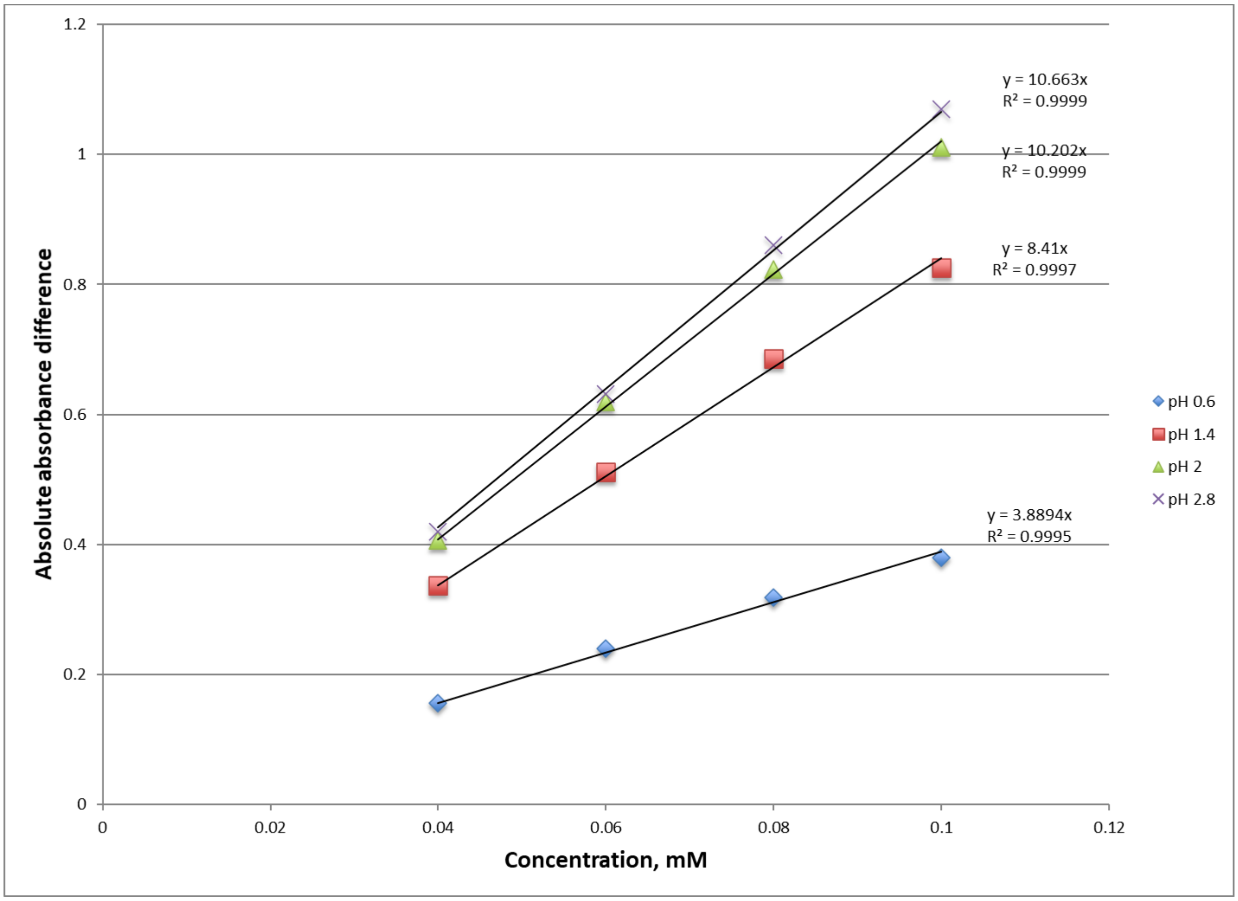

Figure S3. Absolute absorbance difference vs. concentration for 4-nitrotenacic acid at the equilibrium $H_3A \leftrightarrows H_2A^-$. The absorbance difference values were obtained at analytical wavelengths 340 nm (maximum) and 286 nm (minimum).

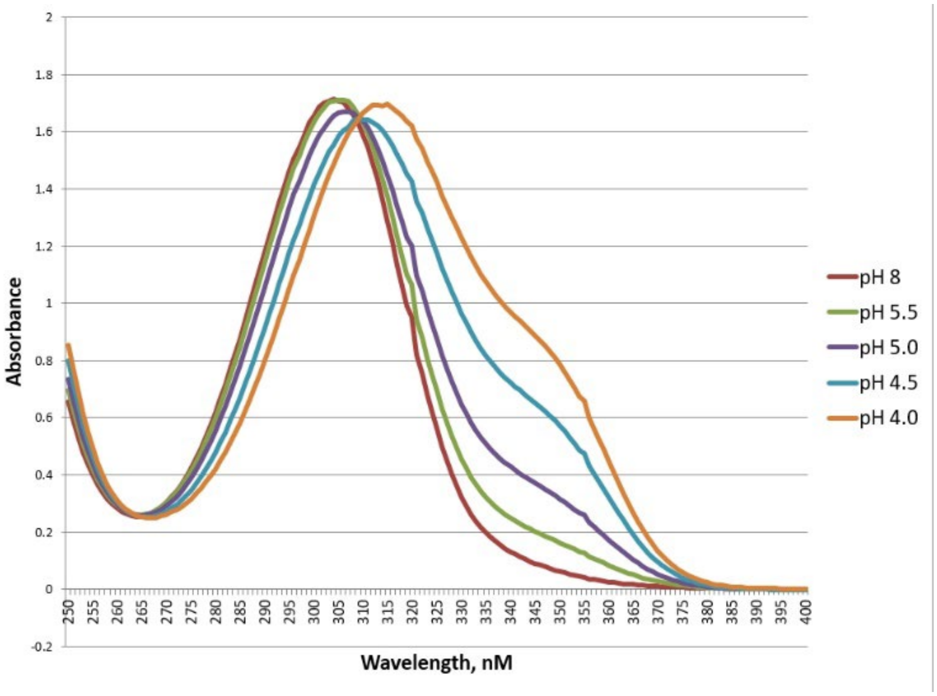

Figure S4. Absorbance spectra of 0.4 mM tenacic acid at equilibrium $H_2A^- \leftrightarrows HA^{2-}$. Similar sets of curves were obtained for 0.1, 0.2 and 0.3 mM samples at the same pH as here.

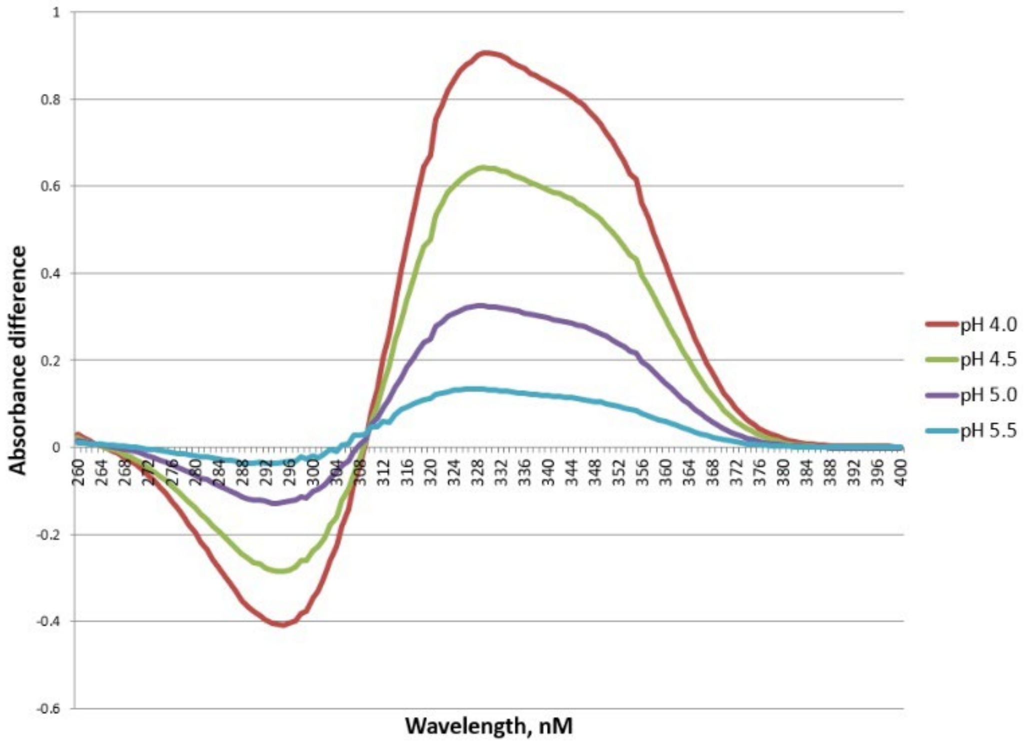

Figure S5. Absorbance difference spectra of 0.4 mM tenacic acid at equilibrium $H_2A^- \leftrightarrows HA^{2-}$. Similar sets of curves were obtained for 0.1, 0.2 and 0.3 mM samples at the same pH as here.

Table S3. Absorbance difference data for determining pK_{a2} of tenacic acid. The reference spectrum was for the sample with pH 8.0. Spectral maximum (Max) = 330 nm; spectral minimum (Min) = 295 nm. The absolute absorbance difference values (ΔA at Max – ΔA at Min) were used for plotting their dependence on concentration and pH.

C, mM		pH 4.0	pH 4.5	pH 5.0	pH 5.5
0.1	ΔA at Max	0.247	0.1755	0.091	0.0273
	ΔA at Min	-0.1205	-0.0808	-0.0336	-0.0212
	ΔA at Max – ΔA at Min	0.3675	0.2563	0.1246	0.0485
0.2	ΔA at Max	0.4399	0.3063	0.1589	0.061
	ΔA at Min	-0.2183	-0.1386	-0.0662	-0.0242
	ΔA at Max – ΔA at Min	0.6582	0.4449	0.2251	0.0852
0.3	ΔA at Max	0.6733	0.477	0.2335	0.0931
	ΔA at Min	-0.3155	-0.2084	-0.106	-0.0417
	ΔA at Max – ΔA at Min	0.9888	0.6854	0.3395	0.1348
0.4	ΔA at Max	0.9066	0.6421	0.3249	0.134
	ΔA at Min	-0.4088	-0.285	-0.1282	-0.0375
	ΔA at Max – ΔA at Min	1.3154	0.9271	0.4531	0.1715

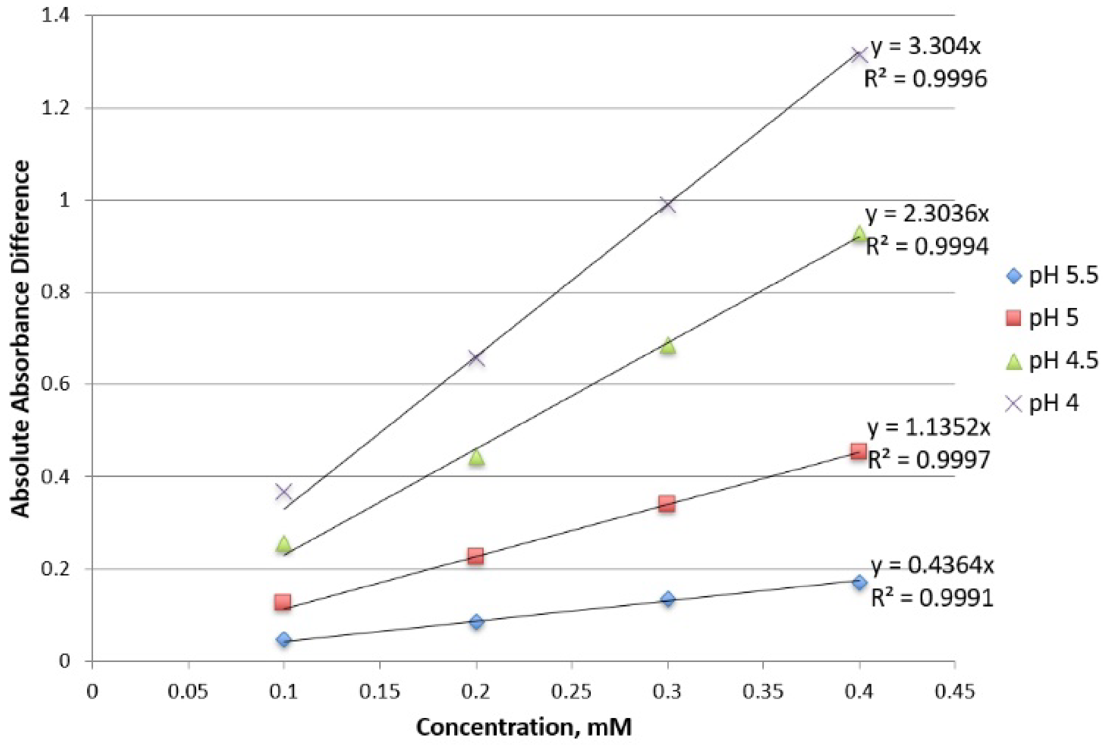

Figure S6. Absolute absorbance difference vs. concentration for tenacic acid at the equilibrium H_2A^- ⇆ HA^{2-}. The absorbance difference values were obtained at analytical wavelengths 330 nm (maximum) and 295 nm (minimum).

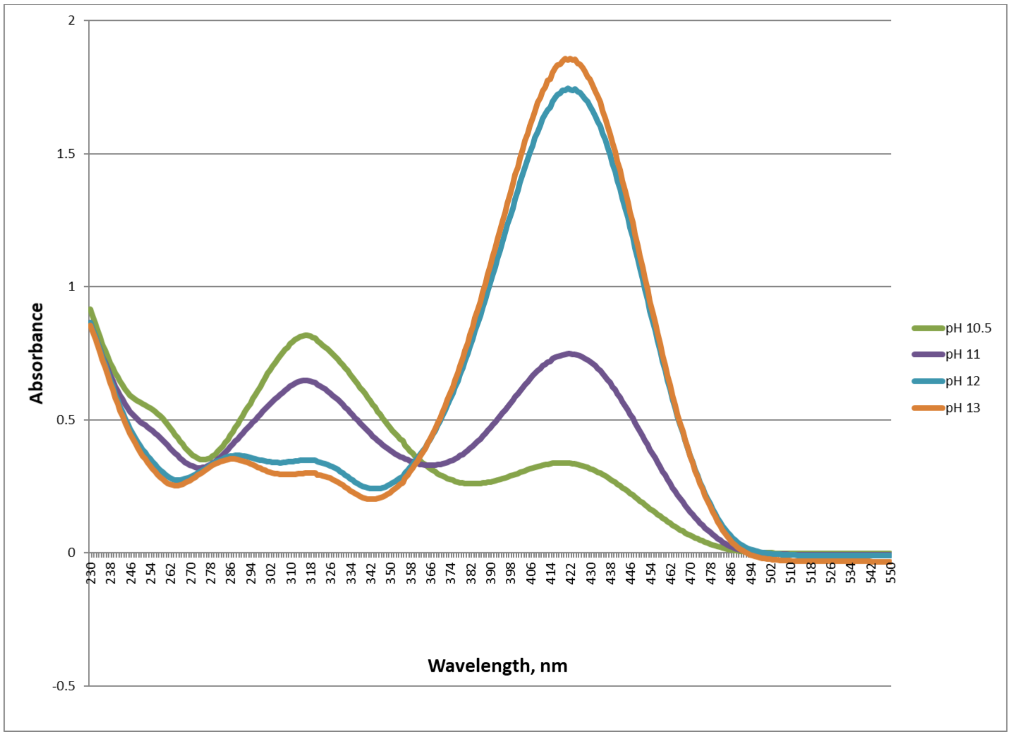

Figure S7. Absorbance spectra of 0.10 mM 4-nitrotenacic acid at equilibrium $H_2A^- \leftrightarrows A^{3-}$. Similar sets of spectra were obtained for 0.04, 0.06 and 0.08 mM samples at the same pH as here.

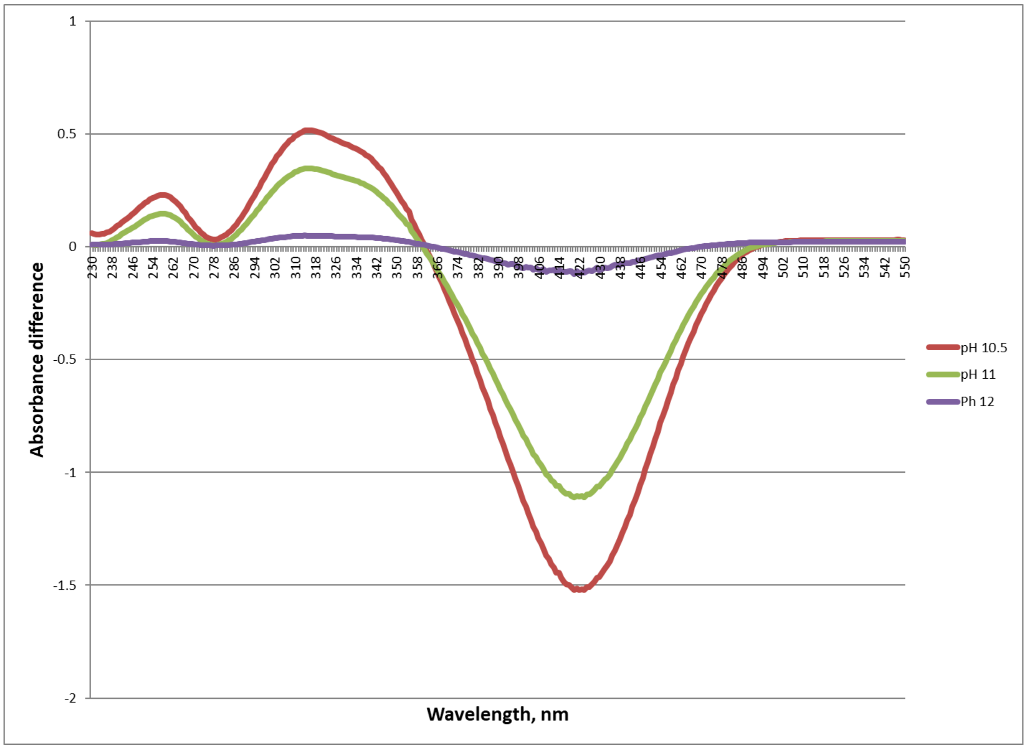

Figure S8. Absorbance difference spectra of 0.10 mM 4-nitrotenacic acid at equilibrium $H_2A^- \leftrightarrows A^{3-}$. Similar sets of curves were obtained for 0.04, 0.06 and 0.08 mM samples at the same pH as here.

Table S4. Absorbance difference data for determining pK_{a3} of 4-nitrotenacic acid. The reference spectrum was for the sample with pH 13. Spectral maximum (Max) = 316 nm; spectral minimum (Min) = 422 nm. The absolute absorbance difference values (ΔA at Max – ΔA at Min) were used for plotting their dependence on concentration and pH.

C, mM		pH 10.5	pH 11.0	pH 12.0
0.04	ΔA at Max	0.2005	0.1322	0.0225
	ΔA at Min	-0.5793	-0.4092	-0.0177
	ΔA at Max – ΔA at Min	0.7798	0.5414	0.0402
0.06	ΔA at Max	0.3032	0.1950	0.0144
	ΔA at Min	-0.9092	-0.6567	-0.0682
	ΔA at Max – ΔA at Min	1.2124	0.8517	0.0826
0.08	ΔA at Max	0.4123	0.2666	0.0378
	ΔA at Min	-1.2251	-0.8970	-0.1074
	ΔA at Max – ΔA at Min	1.6374	1.1636	0.1452
0.10	ΔA at Max	0.5172	0.3479	0.0493
	ΔA at Min	-1.5198	-1.1086	-0.1164
	ΔA at Max – ΔA at Min	2.037	1.4565	0.1657

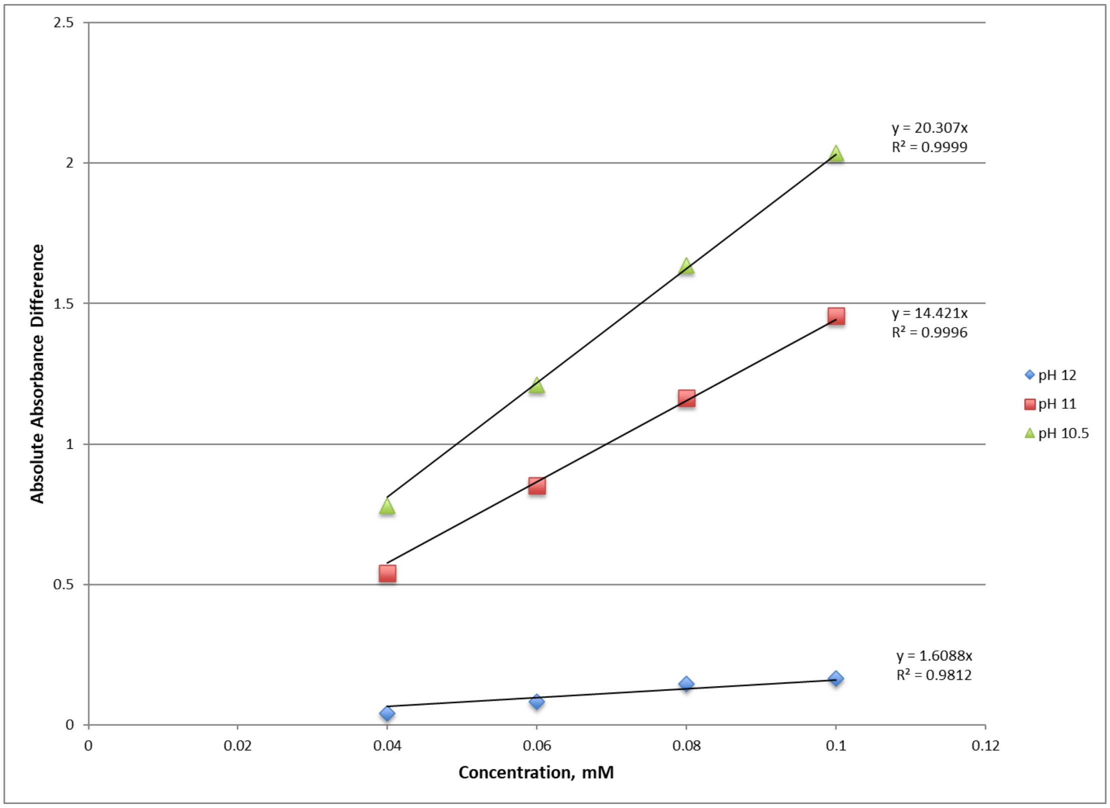

Figure S9. Absolute absorbance difference vs. concentration for 4-nitrotenacic acid at the equilibrium $H_2A^- \leftrightarrows A^{3-}$. The absorbance difference values were obtained at analytical wavelengths 316 nm (maximum) and 422 nm (minimum).

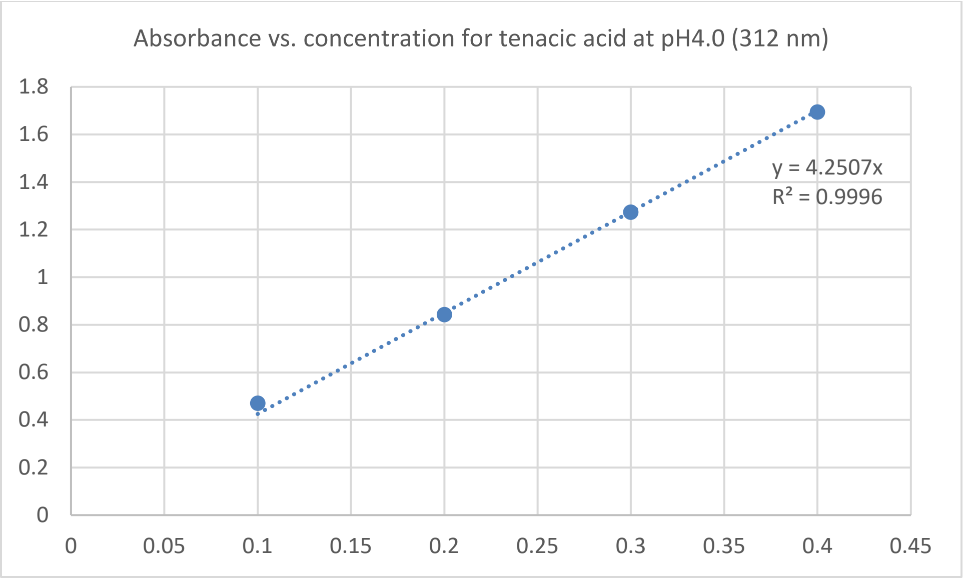

Figure S10. Absorbance vs. concentration for tenacic acid at pH 4.0 and at 312 nm maximum.

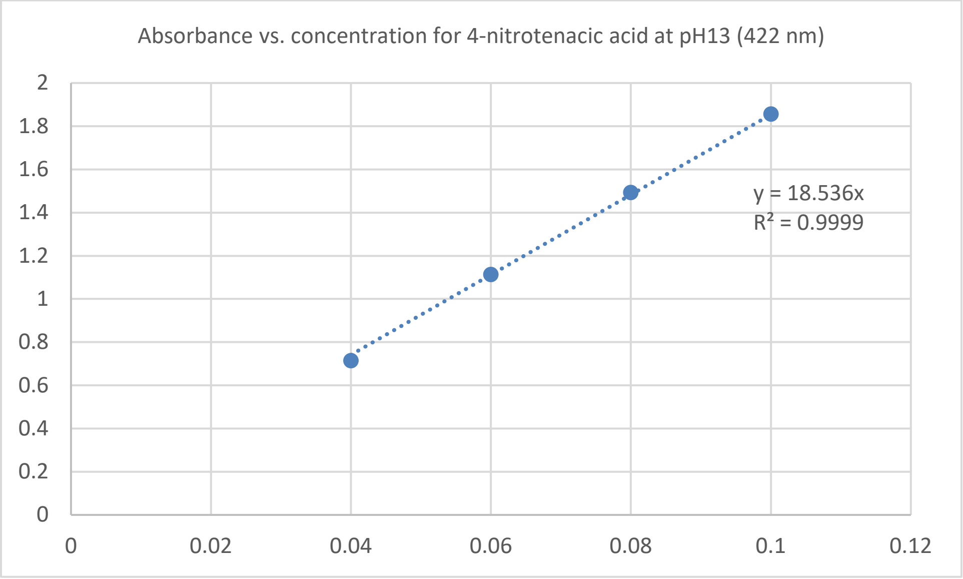

Figure S11. Absorbance vs. concentration for 4-nitrotenacic acid at pH 13 and at 422 nm maximum.

Potentiometric titration method

The potentiometric titration method could directly give the values of pK_{a2} only, because pK_{a1} and pK_{a3} were too low and too high, respectively, for the working range of the potentiometer. For pK_{a3}, the following indirect method for the titration data treatment was used. After the second equivalence point in the titration experiment, the reaction $HA^{2-} + OH^- \rightarrow A^{3-} + H_2O$ takes place, however it does not go to completion because of the too high pK_{a3} value. The titration was continued up to the point where the 3-rd equivalent of base was added, and pH at this point was determined. An imaginary pH was computed, assuming that the acid being analyzed was diprotic, so that the base added after the 2-nd equivalence point, did not react with anything. The difference $\Delta[OH^-]$ between the actual $[OH^-]$ and the imaginary $[OH^-]$ was computed. This difference is attributed to the reaction $HA^{2-} + OH^- \rightarrow A^{3-} + H_2O$, so that $[A^{3-}] = \Delta[OH^-]$. Next, the equilibrium concentration of the dianion was determined: $[HA^{2-}] = [H_3A]_{Total} - [A^{3-}]$. These values and $[H^+]$ at the final point were used to compute the pK_{a3}.

For pK_{a1} of the nitro-acid (2), an indirect method was used as well. Its solubility (17.5 g/L at 22°C) was high enough for this titration. From the titration curve, the pH at the midway before the 1-st equivalence point was determined. One might think that $[H_2A^-] = [H_3A]_{Eq}$ at this point, so pH = pK_{a1}, however since acid being analyzed is relatively strong, $[H_2A^-] > [H_3A]_{Eq}$. In order to determine the actual concentrations of $[H_2A^-]$ and $[H_3A]_{Eq}$ at this point, an indirect method was used. Assuming that acid being analyzed is strong and the equilibrium $[H_3A]_{Eq} = 0$, the imaginary pH at the midway point was computed. The difference $\Delta[H^+]$ between actual $[H^+]$ and imaginary $[H^+]$ was computed, which gave $\Delta[H^+] = [H_3A]_{Eq}$, and finally $[H_2A^-] = [H_3A]_{Total} - [H_3A]_{Eq}$. These values were used for computing the pK_{a1} of the nitro-acid (2).
The results obtained for both acids are shown in Table S5.

Table S5 The pK_a values for both 1 and 2 determined by potentiometric method.

	tenacic acid	4-nitrotenacic acid
pK_{a1}	-	0.72
pK_{a2}	4.72	4.23
pK_{a3}	11.11	10.69
